# Supplementary figures and images for: Signature identification of relapse-related overall survival of early lung adenocarcinoma after radical surgery
Source: PeerJ. 2021 Aug 5;9:e11923. doi: 10.7717/peerj.11923 (PMC8349519; doi:10.7717/peerj.11923)

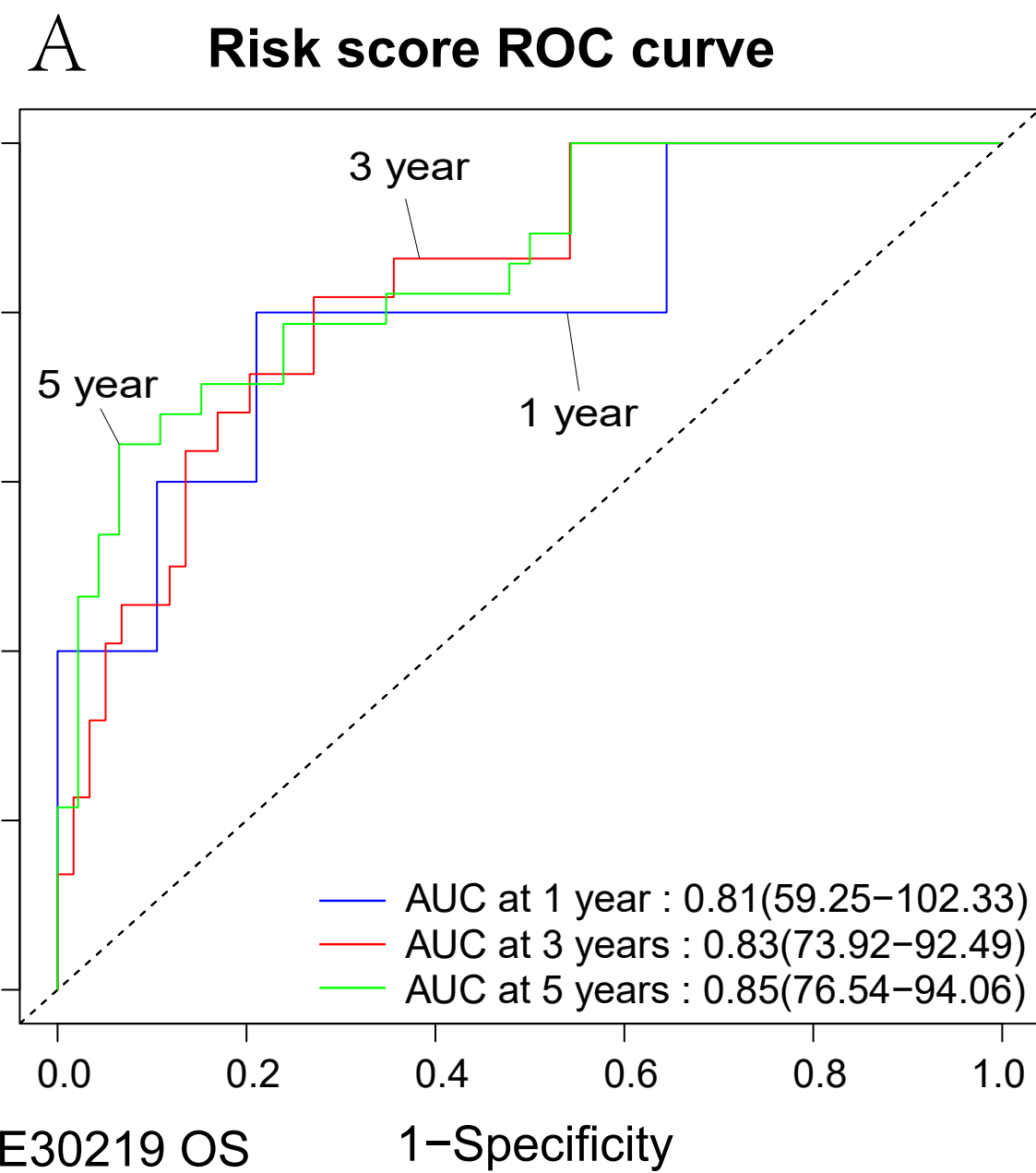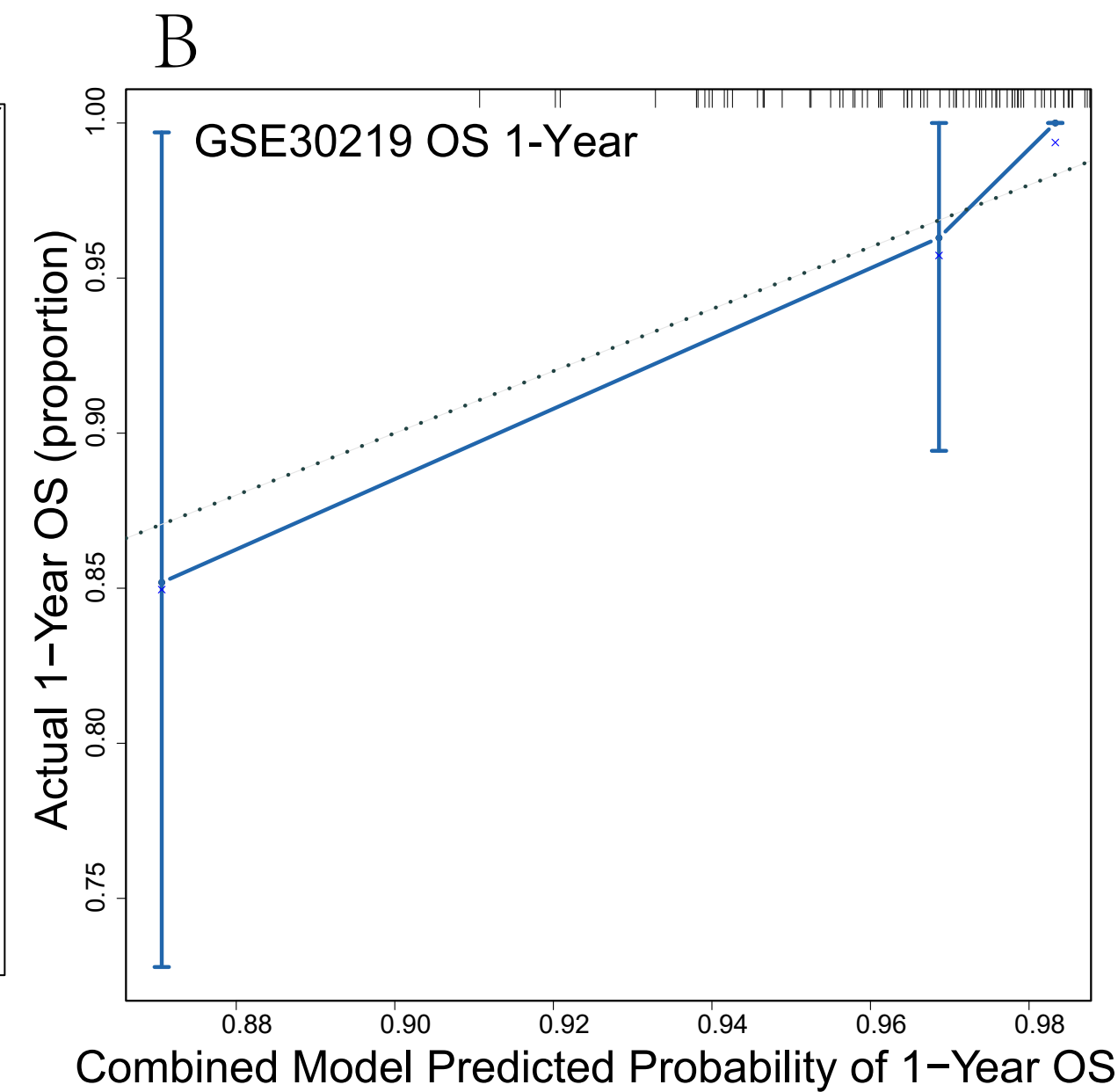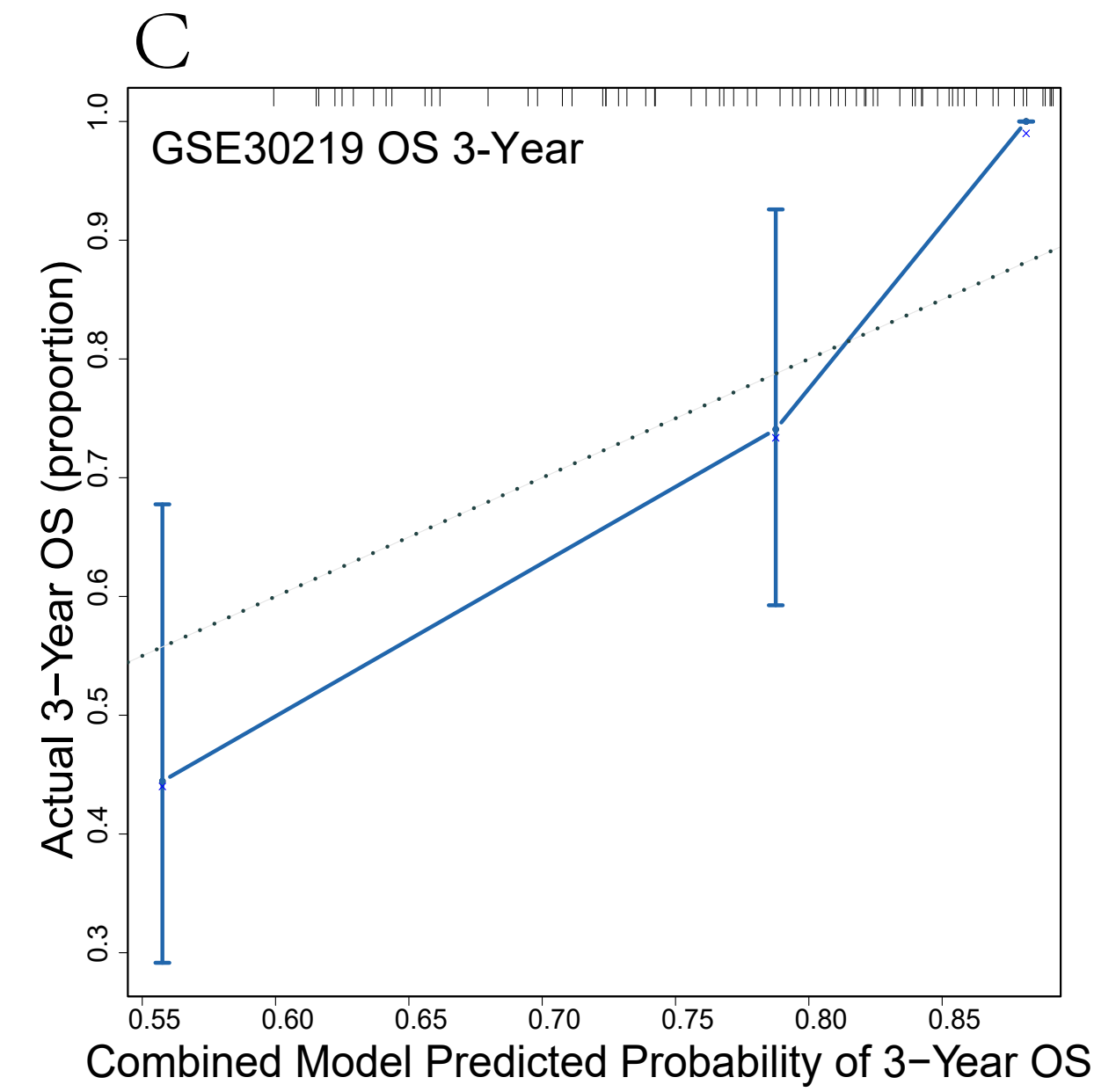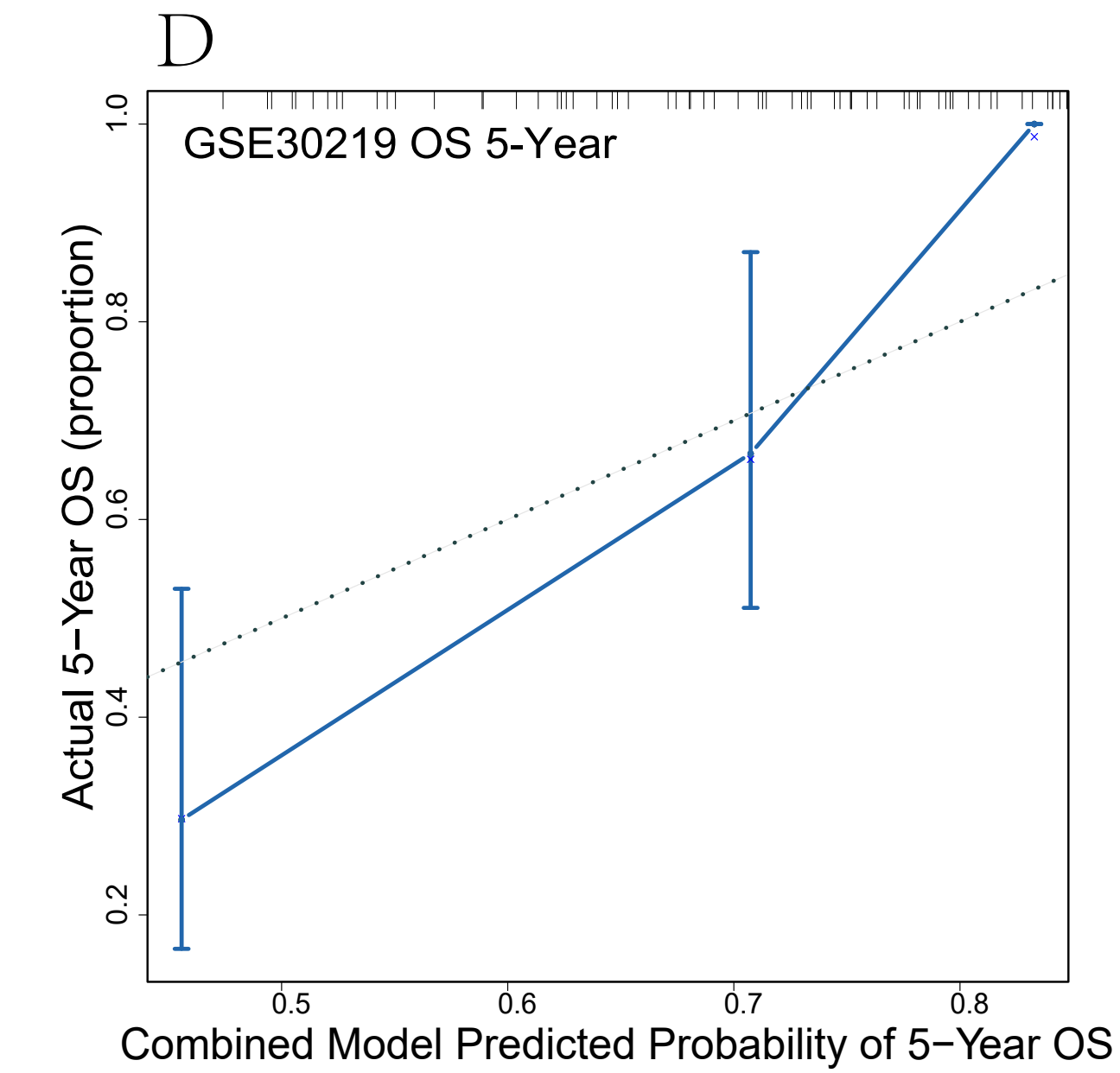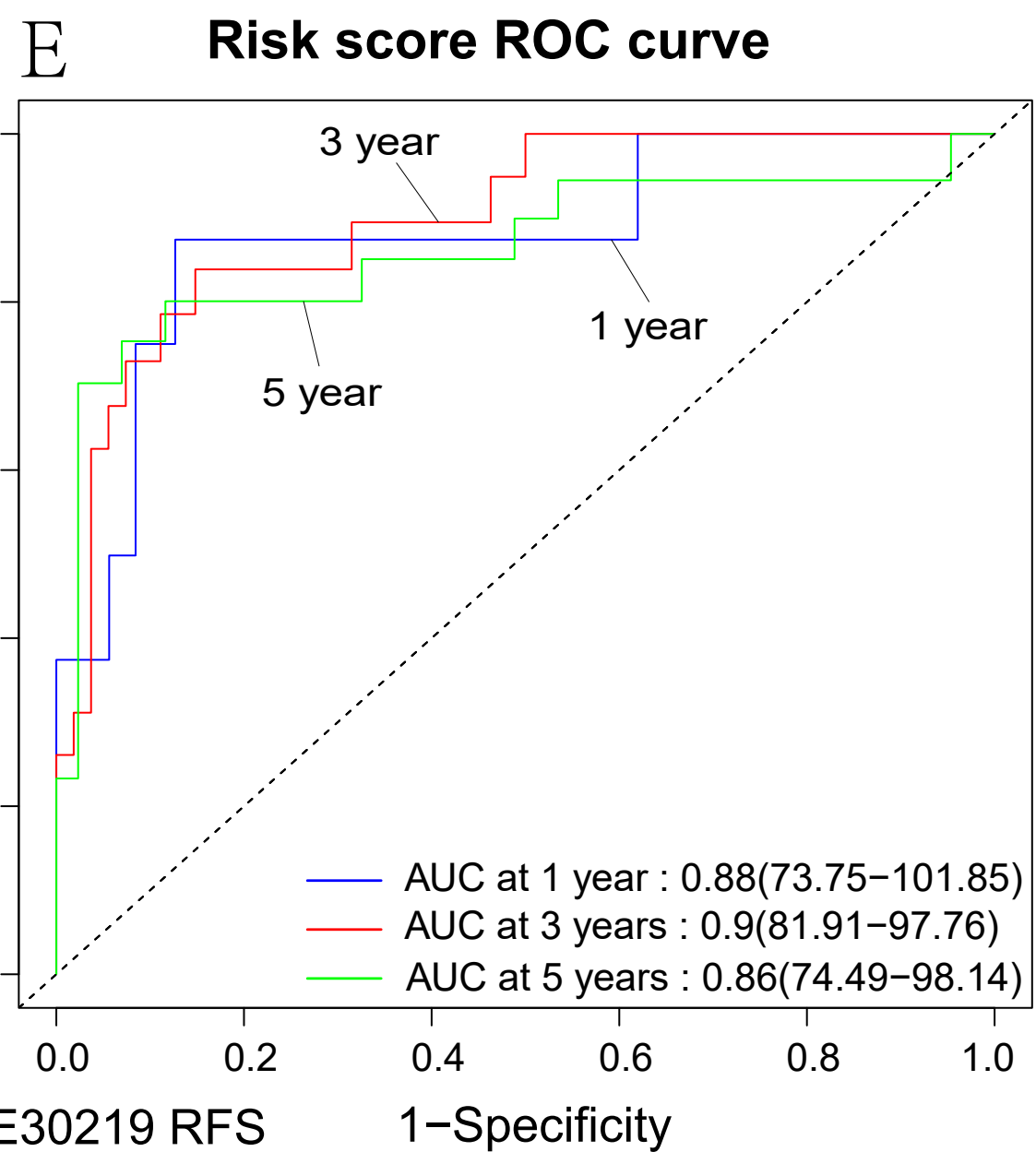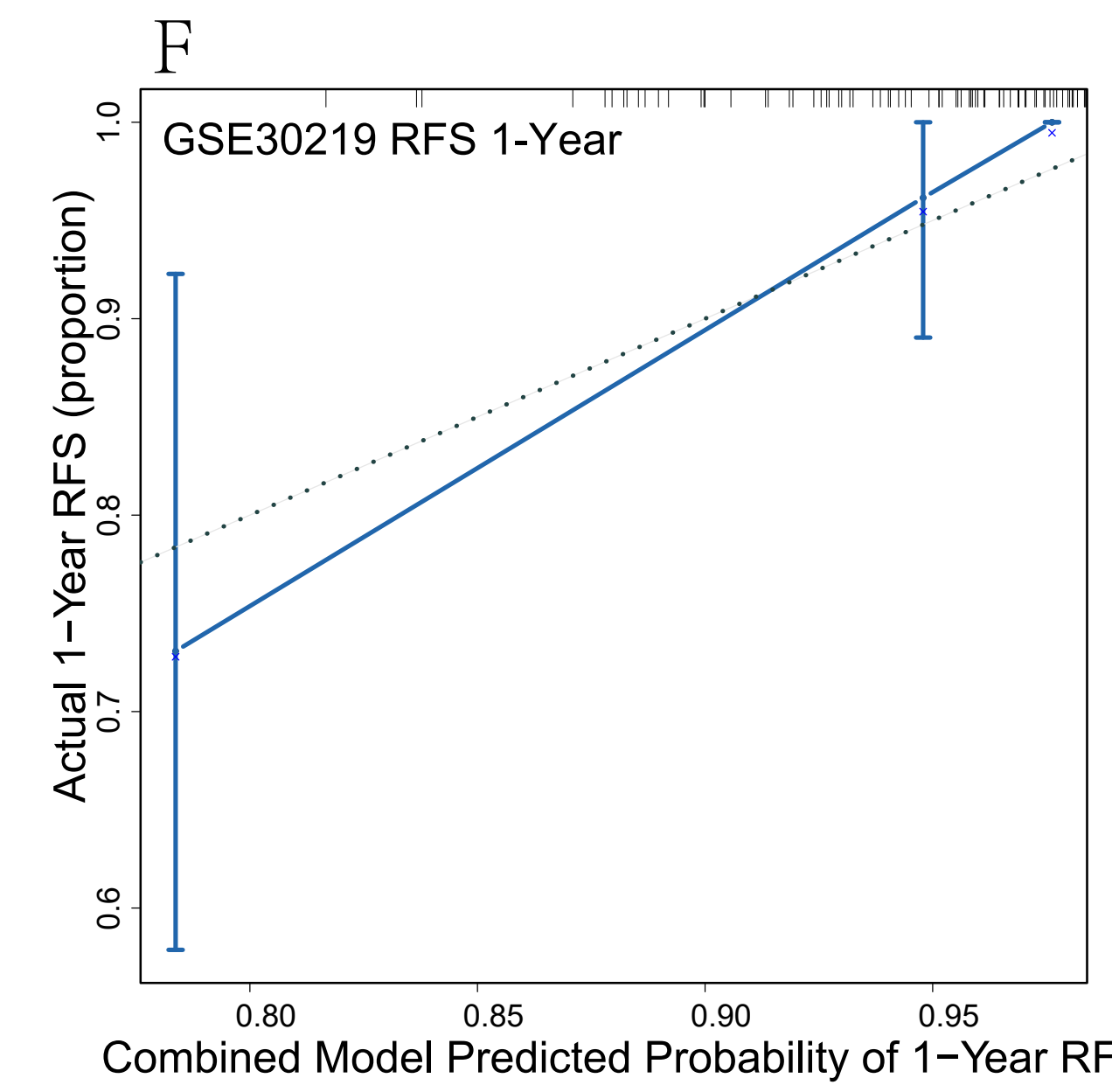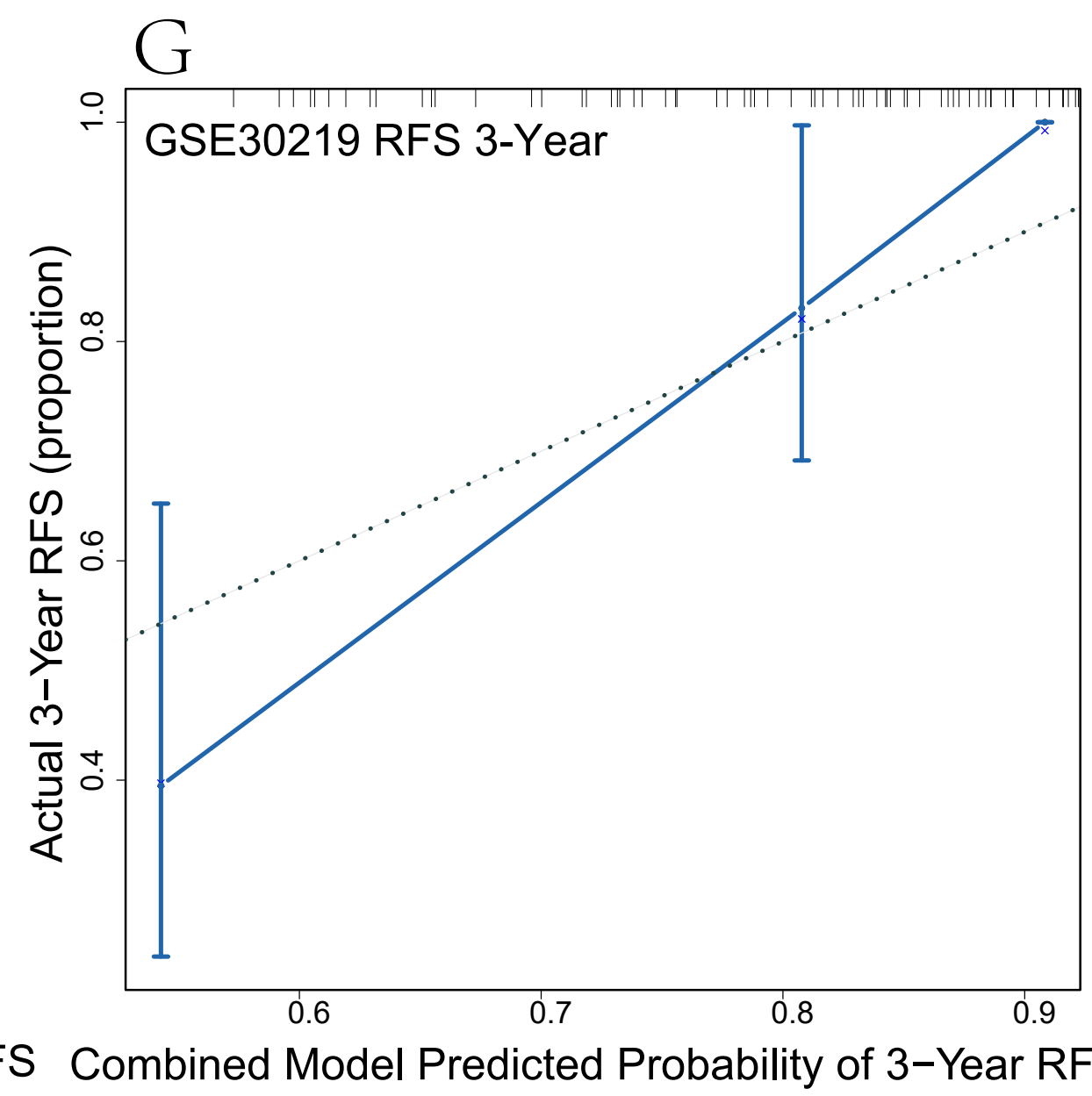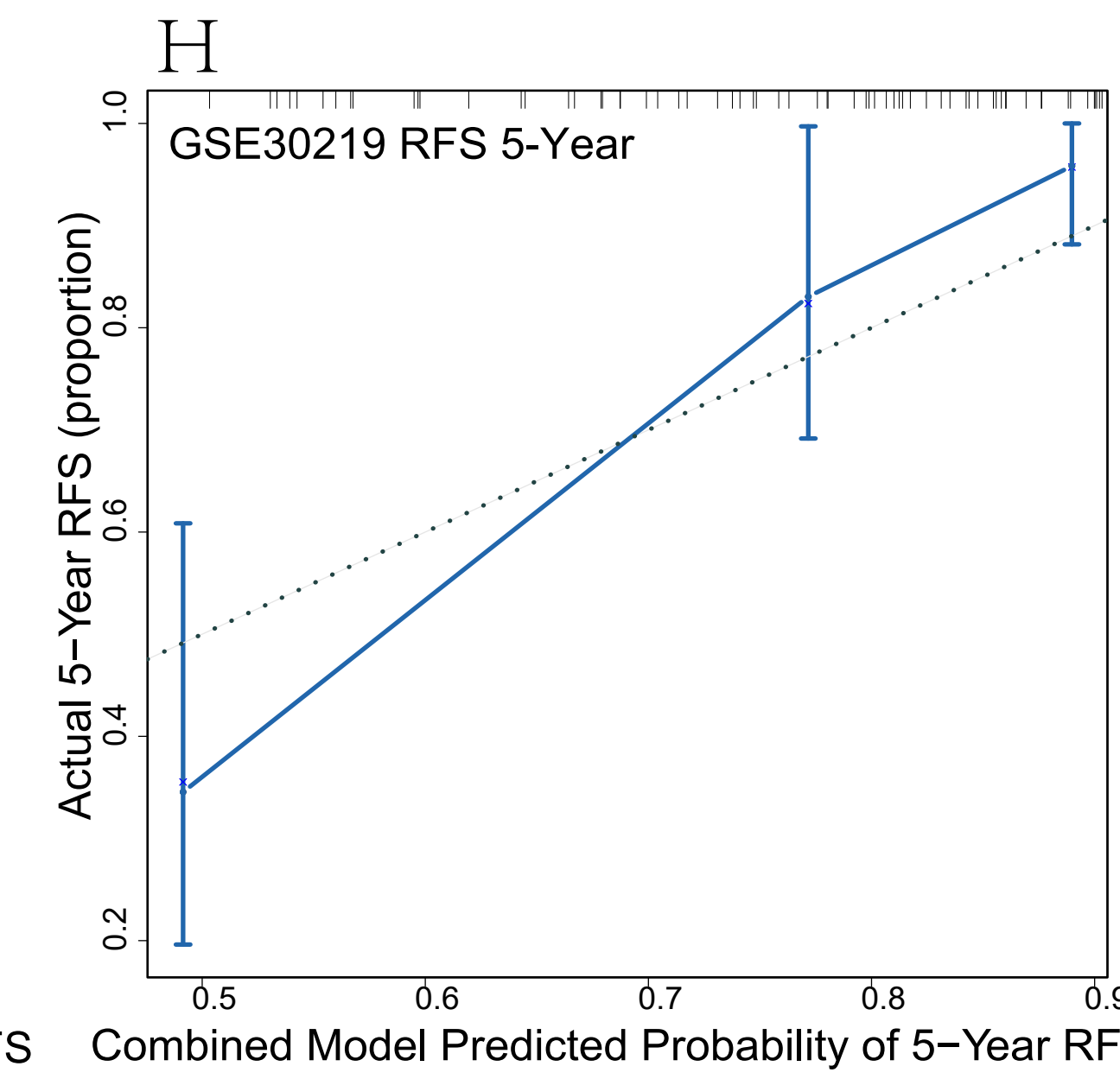

Supplement: Supplemental Information 2 [file peerj-09-11923-s002.pdf]

**Normalization of GSE31210**


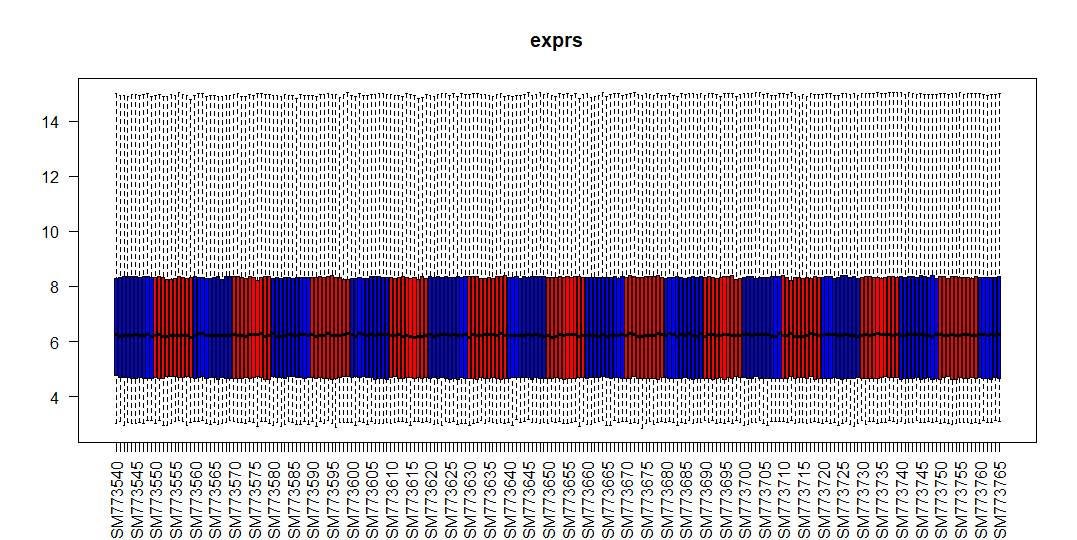

Supplement: Supplemental Information 3 [file peerj-09-11923-s003.docx]
